# Reviewer acknowledgment 2014

**DOI:** 10.1186/s40168-015-0068-7

**Published:** 2015-02-21

**Authors:** Jacques Ravel, K Eric Wommack

**Affiliations:** Department of Microbiology and Immunology, University of Maryland School of Medicine, Institute for Genome Sciences (IGS), Baltimore, Maryland USA; Delaware Biotechnology Institute, University of Delaware, Newark, Delaware USA

## Abstract

We are deeply grateful to all of the names listed below, who reviewed manuscripts submitted to *Microbiome* in 2014. We hope that you all will continue to support the journal as a reviewer or, better yet, as a contributing author.

**Kjersti Aagaard**

United States of America

**Katherine Amato**

United States of America

**Florent Angly**

Australia

**Manimozhiyan Arumugam**

Denmark

**Harsh Bais**

United States of America

**Albert Barberan**

United States of America

**Clara Belzer**

Netherlands

**Jennifer Biddle**

United States of America

**Debby Bogaert**

Netherlands

**Homer Boushey**

United States of America

**Andreas Brune**

Germany

**Frederic Bushman**

United States of America

**Katy Califf**

United States of America

**Ian Carroll**

United States of America

**Frank Chervenak**

United States of America

**Erika Claud**

United States of America

**Maria Carmen Collado**

Spain

**Pierre Cornelis**

Belgium

**Minucci Daria**

Italy

**Suzanne Devkota**

United States of America

**Robert Dyer**

United States of America

**Najwa El-Nachef**

United States of America

**John Erb-Downward**

United States of America

**James Foster**

United States of America

**W. Florian Fricke**

Germany

**Kei Fujimura**

United States of America

**Pawel Gajer**

United States of America

**Jack Gilbert**

United States of America

**Steven Gill**

United States of America

**Greg Gloor**

Canada

**Michael Gorin**

United States of America

**Ann Griffen**

United States of America

**Brian Haas**

United States of America

**Yiping Han**

United States of America

**Robert Hettich**

United States of America

**Markus Hilty**

Switzerland

**Lucas Hoffman**

United States of America

**Deborah Hogan**

United States of America

**Ian Holmes**

United States of America

**Yuichi Hongoh**

Japan

**Michael Imelfort**

Australia

**Ian Jeffery**

Ireland

**Christian Jobin**

United States of America

**Hannah Jones**

United Kingdom

**John Kelly**

United States of America

**Mogens Kilian**

Denmark

**Claudia Knief**

Germany

**Sergey Konstantinov**

Netherlands

**Omry Koren**

United States of America

**Purnima Kumar**

United States of America

**Vladimir Lazarevic**

Switzerland

**Francois Lebreton**

United States of America

**Charles Lee**

New Zealand

**Katherine Lemon**

United States of America

**Catherine Ley**

United States of America

**Huilin Li**

United States of America

**Mark Liles**

United States of America

**John LiPuma**

United States of America

**Nathan Lo**

Australia

**Ramiro Logares**

Spain

**Nicholas Loman**

United Kingdom

**Petra Louis**

United Kingdom

**Susan Lynch**

United States of America

**Bing Ma**

United States of America

**Volker Mai**

United States of America

**Julian Marchesi**

United Kingdom

**Julia Maresca**

United States of America

**David H. Martin**

United States of America

**Rocio Martin**

Netherlands

**Emmanuel F. Mongodin**

United States of America

**Michael Morowitz**

United States of America

**Ardythe Morrow**

United States of America

**Tanya Murphy**

United States of America

**Kenneth Nickerson**

United States of America

**Dennis S. Nielsen**

Denmark

**William Nierman**

United States of America

**Orla O'Sullivan**

Ireland

**George O'Toole**

United States of America

**John Parkinson**

Canada

**Rembert Pieper**

United States of America

**Jose Miguel Ponciano**

United States of America

**Thomas Rattei**

Austria

**John Rawls**

United States of America

**Mary Regan**

United States of America

**Juan Miguel Rodriguez**

Spain

**Gail Rosen**

United States of America

**Bernard Rousseau**

United States of America

**Susannah Salter**

United Kingdom

**Karen Schwarzberg**

United States of America

**Cynthia Sears**

United States of America

**Julia Segre**

United States of America

**Thomas Sharpton**

United States of America

**Jason Stajich**

United States of America

**Yijun Sun**

United States of America

**Eric Triplett**

United States of America

**Silvia Turroni**

Italy

**Chris van der Gast**

United Kingdom

**Christian von Mering**

Switzerland

**William Wade**

United Kingdom

**Alan Walker**

United Kingdom

**Jens Walter**

Canada

**Hung Winn**

United States of America

**Dongying Wu**

United States of America

**Yanbao Yu**

United States of America

**Egija Zaura**

Netherlands

